# Biphasic expression of thyroid hormone receptor TRβ1 in mammalian retina and anterior ocular tissues

**DOI:** 10.3389/fendo.2023.1174600

**Published:** 2023-03-23

**Authors:** Lily Ng, Hong Liu, Ye Liu, Douglas Forrest

**Affiliations:** National Institute of Diabetes and Digestive and Kidney Diseases, Laboratory of Endocrinology and Receptor Biology, National Institutes of Health, Bethesda, MD, United States

**Keywords:** thyroid hormone receptor, *THRB* gene, retina, anterior eye, neurodevelopment

## Abstract

The retina is increasingly recognized as a target of thyroid hormone. We previously reported critical functions for thyroid hormone receptor TRβ2, encoded by *Thrb*, in cones, the photoreceptors that mediate color vision. TRβ1, another *Thrb* receptor isoform, is widely expressed in other tissues but little studied in the retina. Here, we investigate these N-terminal isoforms by RNA-sequencing analysis and reveal a striking biphasic profile for TRβ1 in mouse and human retina. In contrast to the early TRβ2 peak, TRβ1 peaks later during retinal maturation or later differentiation of human retinal organoids. This switch in receptor expression profiles was confirmed using *lacZ* reporter mice. TRβ1 localized in cones, amacrine cells and ganglion cells in contrast to the restricted expression of TRβ2 in cones. Intriguingly, TRβ1 was also detected in the retinal pigmented epithelium and in anterior structures in the ciliary margin zone, ciliary body and iris, suggesting novel functions in non-retinal eye tissues. Although TRβ1 was detected in cones, TRβ1-knockout mice displayed only minor changes in opsin photopigment expression and normal electroretinogram responses. Our results suggest that strikingly different temporal and cell-specific controls over TRβ1 and TRβ2 expression may underlie thyroid hormone actions in a range of ocular cell types. The TRβ1 expression pattern suggests novel functions in retinal and non-neural ocular tissues.

## Introduction

A growing body of evidence indicates the sensitivity of the mammalian retina to thyroid hormone and the potential for retinal dysfunction in thyroid disorders. Genetic studies have revealed particularly critical functions in cone photoreceptors, the specialized cells that mediate color vision and daylight vision. Color vision is mediated by cone populations with opsin photopigments for response to different regions of the light spectrum, usually medium-long (M, “green”) or short (S, “blue”) wavelength regions in mammals (1, 2). Mice deleted for thyroid hormone receptor TRβ2, encoded by *Thrb*, lack M opsin resulting in a form of monochromatic color-blindness (3). These findings reflect a key role for TRβ2 in promoting M and S cone diversity from cone precursors with a default S opsin identity (4, 5). Mutations of the *THRB* gene in human resistance to thyroid hormone have been associated with monochromacy and impaired responses to medium-long wavelength light (6–8). Mutation of *THRB* in human retinal organoids impairs expression of opsins for medium-long wavelength responses (9), suggesting conserved functions for the *Thrb* gene in the mammalian retina.

Thyroid hormone also influences photoreceptor differentiation and survival. Hypothyroidism in rodents impairs M opsin expression (10–12) and responses to green wavelength light (13). Hyperthyroidism can alter opsin expression (14) but excesses cause cone cell death in mice (15, 16). Human population studies suggest an association of high levels of thyroxine (T4), the major circulating form of thyroid hormone, with age-related macular degeneration (17, 18), a disorder of the retinal pigmented epithelium that leads to deterioration of photoreceptors. Inhibition of thyroid hormone signaling can reduce the loss of photoreceptors in mouse models of retinal degeneration (15, 19) or macular degeneration (20). Accordingly, studies to elucidate the receptor-mediated basis for thyroid hormone action in the retina might suggest new therapeutic approaches for retinal disease.

Mapping of receptor expression patterns has been instrumental in identifying cellular targets for thyroid hormone. TRβ2 and TRβ1 isoforms encoded by *Thrb* differ in their N-termini but share common DNA binding and ligand binding domains. TRβ1 is widely expressed including in the pituitary, brain, liver and cochlea (21–23) but has been little studied in the retina. Differential expression of isoforms in the retina was first indicated in the chick embryo by *in situ* hybridization with specific N-terminal probes: TRβ2 localized in the outer nuclear (photoreceptor) layer whereas a TRβ1-like isoform appeared later in the outer nuclear layer and inner nuclear (interneuron) layer (24). However, previous *in situ* hybridization analyses lacked cell type resolution. Subsequently, TRβ2 expression was localized in cones using knockin or transgenic reporters in mice (3, 25) and transgenic reporters in avian and fish species (26, 27). The TRβ1 cellular expression pattern is undefined. RNA-sequencing (RNA-seq) is now common for gene expression studies of the retina or retinal organoid cultures as a model system [e.g., see refs (9, 28, 29).]. However, standard RNA-seq analyses yield reads for the total *Thrb* gene and do not distinguish TRβ1- and TRβ2-specific 5’ exons, which lie > 6 kb upstream in the mRNA (30). In single cell analyses, the limitations are compounded by the very small amounts of input RNA (4, 31).

We have tested the hypothesis that TRβ1 has a role in cones or other retinal cell types by determination of the TRβ1 expression profile in mouse and human retina using customized RNA-seq analysis. We localized cellular expression of TRβ1 using a knockin *lacZ* reporter (23) and investigated cone phenotypes in TRβ1-knockout mice. The results show a unique pattern of TRβ1 expression that differs strikingly from that of TRβ2, and suggest versatile roles for the *Thrb* gene in the retina and other non-neural tissues of the eye.

## Materials and methods

### Mouse strains

Tissue expression of TRβ1 was investigated in *Thrb* ^b1^ lacZ reporter mice (23) that express β-galactosidase instead of TRβ1 and which in the homozygous state represent a knockout of TRβ1. TRβ2 expression was investigated using a *Thrb* ^b2^ lacZ knockin (3); homozygotes (*Thrb* ^b2/b2^) were used to enhance detection of signals. Phenotypic analyses were performed on homozygotes of each strain representing knockouts of TRβ1 and TRβ2, respectively. Both strains were back-crossed to a C57BL/6J background for ~10 generations. Genotyping was performed by PCR as described (3, 23). Experiments followed approved protocols at NIDDK at the National Institutes of Health.

### Library construction and RNA-seq analysis

Total RNA was prepared from pooled retinas of C57BL/6J mice (The Jackson Lab, cat # 000664). Each pool represented 4 - 6 embryos (8 - 12 retinas) or ≥ 3 postnatal mice (≥ 6 retinas), except at P60, two samples represented pools of 3 mice (6 retinas) and four samples represented individual mice (both retinas per mouse). At postnatal ages, males were selected to provide datasets of a defined sex. For practical reasons, for embryos and P1 neonates, when sex could not be determined visually at time of collection, samples included mixed sexes; larger pools were required at these early stages as tissue amounts were smaller. RNA was prepared using TRIzol (ThermoFisher) and RNeasy Micro kit (Qiagen, cat# 74004) isolation. Each RNA-seq library was constructed from ~250 ng of purified RNA using SMARTer total RNA Sample Prep kit (TakaraBio, cat# 634874). Libraries were sequenced on an Illumina HiSeq-2500 instrument at the NIDDK Genomics Facility. For each library, ~20 million single-end 50 base reads were collected, then converted using bcl2fastq (version 2) into fastq files and aligned on (GRCm38/mm10) with STAR (version 2.7.3a).

**Figure 1:**
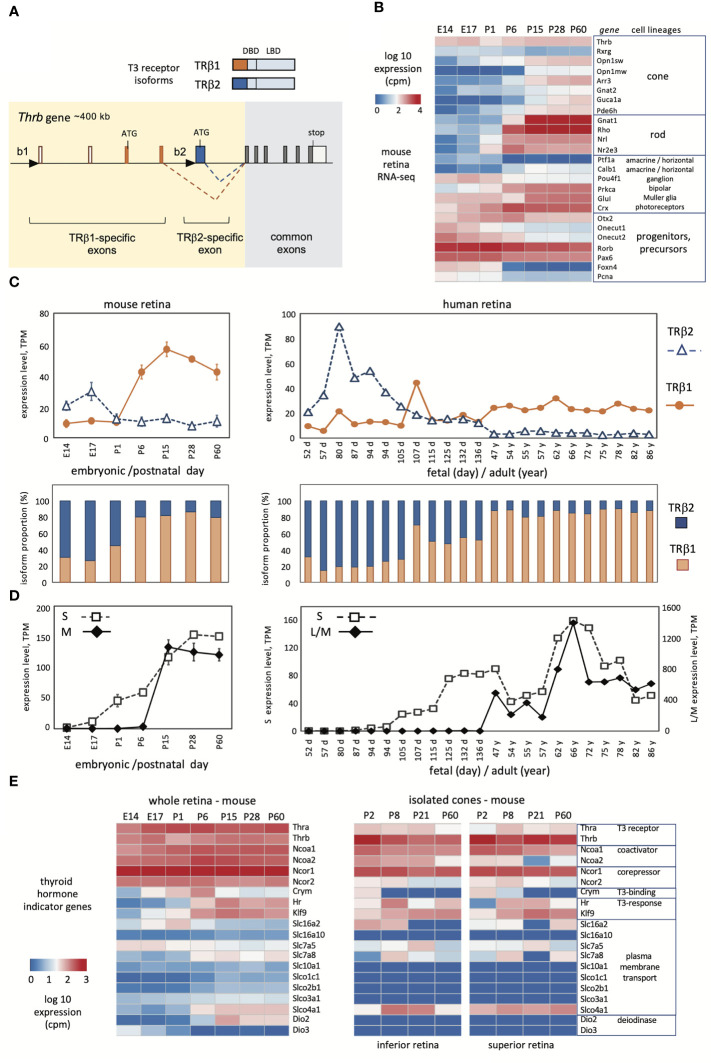
Developmental profiles of TRβ1 and TRβ2 in mouse and human retina. **(A)**, Mouse *Thrb* gene showing TRβ1- and TRβ2-specific 5’-exons and promoters (black triangles) and receptor products above. DBD, DNA binding domain, LBD, ligand binding domain. **(B)**, Overview of retinal lineage markers in RNA-seq datasets in mice. Groups, n = 3 to 6; mean ± S.D., cutoff average = 10 cpm. **(C)**, Developmental profiles of TRβ1 and TRβ2 in mouse (*left*) and human retina (*right*). Mouse RNA-seq datapoints represent groups, mean ± S.D.; human datapoints, individual samples. **(D)**, Cone opsin expression in the same RNA-seq datasets; S opsin and M opsin (mouse) or L/M opsins (human). The human L/M opsin curve represents reads from both genes in the *OPN1LW-OPN1MW* gene cluster. **(E)**, Representative indicator genes for thyroid hormone signaling in mouse whole retina (*left*) or isolated cones (*right*; cones from superior or inferior regions).

Dataset analyses: Transcripts were analyzed in bam files using STAR (v2.6.0c, https://github.com/alexdobin/STAR) as counts per million reads (cpm), or quantified in fastq files using Kallisto (version 0.46.1, https://pachterlab.github.io/kallisto/) as transcript per million reads (TPM). Samples at a given age were highly consistent as shown by a principal component analysis, in which the major source of variance was contributed by developmental age (87.9% for the first principal component). To analyze TRβ1 and TRβ2 isoforms, customized reference indices were created for four defined TRβ1-specific exons (see **Figure 1A**) and the single TRβ2-specific exon after removal of the total *Thrb* gene exons from the reference genome index. The isoform-specific indices were analyzed in STAR or Kallisto programs. Total *Thrb* reads were calculated for a standard *Thrb* whole gene reference index based on NCBI or ENSEMBL databases using STAR or Kallisto. Gene expression heatmaps were generated using gplots in R version 4.2.1 (https://cran.r-project.org/) for these retinal datasets and previously reported isolated cone datasets (groups of 21 - 30 cells/age) (4).

## Human retinal RNA-seq analysis

Fetal datasets provided by Drs. A. Swaroop and T. Reh (32) were obtained from the NEI-Commons at the National Eye Institute (https://neicommons.nei.nih.gov/#/dataSearch) (GEO accession GSE104827). Adult retina datasets representing peripheral retinal punches provided by Drs. A. Swaroop, M.J. Brooks and K. Kaya (33) were obtained from GEO (GSE115828). For PEN8E iPSC-derived retinal organoids (NEI-001 control A) (34), groups included 2 or 3 samples (GSE129104). The “60 day” stage represents 2 samples at 60 days and one at 67 days in culture. For H9 retinal organoids (35), groups included two samples per stage. For analyses of human *THRB* isoforms, customized indices were created for the TRβ1- and TRβ2-specific exons equivalent to the exons used for the mouse gene, and separately for *THRB* common exons, using STAR or Kallisto programs. Gene expression heatmaps were generated from human retinal and retinal organoid datasets using R version 4.2.1 (https://cran.r-project.org/).

### TRβ1 and TRβ2 cDNA isolation

Total RNA from PEN8E retinal organoids (34) was kindly provided by Anand Swaroop and Zepeng Qu (National Eye Institute, NIH). RNA pooled from organoids at 90 and 120 day stages was reverse-transcribed into cDNA using the SMARTer RACE 5’/3’ kit (TakaraBio, Cat# 634858). Specific primers for PCR-amplification of full-length TRβ1 or TRβ2 coding cDNAs were based on *THRB* genomic exon sequences. Specific 5’ primer for TRβ1: Fb1, TTG CAT GAA TAA TGT GAG TGC; specific 5’ primer for TRβ2: Fb2, TAT GCT TCT CTG CGT ATA TGC CCA GC; common 3’-end reverse primer, Rc, CCA AAT AAT CCC TCC CAA CAC.

### Quantitative PCR analysis

Analysis of gene expression followed described procedures (4). Briefly, total RNA was extracted using RNeasy Plus mini kit (Qiagen, Cat #74136) from whole retina or sub-dissected pieces of superior or inferior retina. First-strand cDNA was synthesized using Superscript IV Reverse Transcriptase kit with oligo dT primers (Thermofisher, Cat #18091050). The qPCR reaction was performed with FastStart Universal SYBR Green Master-Mix (Roche; Cat #04913914001) on a StepOne or QuantStudio 3 instrument with analysis using software provided by the manufacturer (ThermoFisher). Relative gene expression levels were quantified using the 2^−ΔΔ^ *C*_T_ method (36) and normalized to Hprt (for photoreceptor gene analysis) or Actb (for TRβ1 and TRβ2 isoform analysis) as internal controls.

*Primer pairs for photoreceptor genes*: Opn1mw-F: CTG GTG AAC TTG GCA GTT GC; Opn1mw-R: AAA TGA TGG CCA GGG ACC AG; Opn1sw-F: ATG CAC TGA TGG TGG TCC TG; Opn1sw-R: CAG ACT CTT GCT GCT GAG CT; Rho-F: TTG GCT GGT CCA GGT ACA TC; Rho-R: GAA TGG TGA AGT GGA CCA CG; Arr3-F: TCA GTA ACA CTG CAG CCT GG; Arr3-R: CAT CCA GGC CTG CAG TTG TA;

Ccdc136-F: TGA GAT GGA GAT TGC CTC GC; Ccdc136-R: TCG TAC TCC GTA GCA GGT GA; Gucy2e-F: AGT CCA CTG GAC TGC CTT AC; Gucy2e-R: CGT GTC CTC AAT ACC CTT GC; Pgc-F: TAG CCT GCC TAC CCT CAC TT; Pgc-R: CCC ACC CTG TTA TTG CCC AT; Kcne2-F: AGG TCT CCT GCA TTG CTC AC; Kcne2-R: TGC CGA TCA TCA CCA TGA GG; Grk1-F: GAG GAG AGA AGG TAG AGA AC; Grk1-R: CCA ACA GCT GCT CAC AGA AG; Hprt-F: TAC CTC ACT GCT TTC CGG AG, Hprt-R: ATC GCT AAT CAC GAC GCT GG;

*Primer pairs for TRβ1 and TRβ2*: Trb1-F: AAT AAG AAG GTC AGA GGG AAT GCC; Trb1-R: CCT GGA TAA GGT GTG GGG AAG TC; Trb2-F: CCT GTA GTT ACC CTG GAA ACC TG; Trb2-R: TAC CCT GTG GCT TTG TCC C; Actb-F: CAC AGC TTC TTT GCA GCT CC; Actb-R: ACC CAT TCC CAC CAT CAC AC;

### Histochemistry and immunostaining

Experiments followed previously described procedures (5). Retinas were fixed in 1% PFA for 1 hour for β-galactosidase histochemistry or 3 hours for immunostaining. Twelve μm-thick cryosections were incubated with 5-bromo-4-chloro-3-indolyl-D-galactopyranoside (xgal)(1 mg/mL) using a β-Galactosidase Reporter Gene Staining kit (Sigma). Images were obtained using a Nikon 80i microscope. At each stage, at least 6 eyes (n ≥ 3 mice) were examined. For immunostaining, sections were incubated with primary antibodies overnight then with secondary antibodies for 1 hour. Images were obtained using a Leica TCS SPE confocal microscope and processed using ImageJ software.

*Primary antibodies* (*target antigen, type, dilution, source, RRID*): TRβ2, rabbit polyclonal, 1:2,000 (37) (RRID : AB_2927439); β-galactosidase, chicken polyclonal, 1:500 (Abcam ab9361, RRID : AB_307210); Arr3, rabbit polyclonal, 1:1,000 (Millipore, AB15282, RRID : AB_1163387); Calbindin, rabbit polyclonal, 1:500 (Millipore, AB 1778, RRID : AB_2068336); RBPMS, rabbit polyclonal, 1:500 (PhosphoSolutions, 1830-RBPMS, RRID : AB_2492225)*;* Calretinin, rabbit polyclonal, 1:500 (Millipore AB 5054, RRID : AB_2068506); S opsin, rabbit polyclonal, 1:500 (Millipore, AB 5407, RRID : AB_177457); M opsin, rabbit polyclonal, 1:1,000 (Millipore AB 5405, RRID : AB_177456); Rho, rabbit polyclonal, 1:500 (Abcam AB40673, RRID : AB_777706). *Secondary antibodies*: Alexa Fluor 488-conjugated anti-chicken IgY, goat polyclonal, 1:500 (Invitrogen, A11039, RRID : AB_2534096); Alexa Fluor 568-conjugated anti-rabbit IgG, goat polyclonal, 1:500 (Invitrogen, A11011, RRID : AB_143157). *Lectin*: Rhodamine Peanut Agglutinin (PNA), 10 μg/ml (Vector Lab, RL-1072, RRID : AB_2336642);

### Electroretinogram analysis

Electroretinogram (ERG) analysis was performed as described (5) on 6 - 8 week old mice anesthetized with ketamine and xylazine (25 and 10 microgram per g body weight, respectively). The ERG was recorded using an Espion Electrophysiology System (Diagnosys LLC) for groups (6 - 8 mice) with approximately equal numbers of males and females.

### Statistical analyses

Statistical significance was evaluated using unpaired two-tailed Student’s t-tests, with significance set at p < 0.05. Analyses were performed with GraphPad Prism version 9 (GraphPad Software). Experimental design was based on previous studies (16, 38). Column graphs for cell counts and qPCR analyses were plotted using GraphPad Prism. The ERG b-wave graphs were plotted using Microsoft XL (version 16.69.1).

### Data availability

RNA-seq datasets of mouse retina generated in this work are available at GEO (GSE224863).

RNA-seq datasets of mouse single photoreceptors are available at GEO (GSE 203481).

Human RNA-seq datasets are available at NEI-Commons (https://neicommons.nei.nih.gov) and at GEO: fetal retinal datasets, GSE104827; adult retinal datasets, GSE115828. Datasets for H9 and PEN8E retinal organoids are at GEO (GSE129104). Human TRβ2 and TRβ1 cDNA sequences are available at GenBank access OQ406274 and OQ406275.

## Results

### TRβ1 and TRβ2 profiles in mouse retinal development

The mammalian *Thrb* gene spans > 400 kb of genomic DNA and includes a complex 5’ region with distinct promoters and exons encoding the specific N-termini of TRβ1 and TRβ2 (**Figure 1A**) (39, 40). The common exons encoding the DNA binding and ligand binding domains, and the TRβ2-specific and TRβ1-specific 5’ coding exons are highly conserved in mammalian species (41). We derived RNA-seq datasets to investigate expression of TRβ isoforms in mouse retina from embryonic day 14 (E14) to adulthood (postnatal day 60, P60). This period spans the neurogenesis of retinal cell types, postnatal differentiation and eye opening (~P13), then functional maturation, as verified by a summary heatmap of selected marker genes for retinal cell lineages (**Figure 1B**). This period encompasses the differentiation of cone photoreceptors. The generation of cone precursors begins by ~E12, and is complete by around birth in mice (42). Cones then mature postnatally and express markers including opsin (*Opn1sw*, *Opn1mw*) and phototransduction genes (e.g., *Arr3*, *Guca1a*).

To distinguish TRβ1 and TRβ2 reads, we created customized reference indices for analysis of TRβ1- and TRβ2-specific exons. The TRβ2 index represented the single TRβ2-specific exon. The TRβ1 index represented four exons: the two TRβ1-specific coding exons, the non-coding exon at the TRβ1 promoter region and the next downstream, non-coding exon consistently found in TRβ1-specific cDNAs (depicted in **Figure 1A**). RNA-sequencing was performed with substantial depth to improve detection of low level mRNAs (~18 million reads/library). We detected TRβ1 at very low levels in embryonic retina, then a postnatal increase and plateau at ~P15 with levels maintained into adulthood (**Figure 1C**, first and second row plots). In contrast, TRβ2 was high at embryonic stages then declined postnatally, consistent with northern (3) and western blot (37) analyses. Analyses of *Thrb* total reads (using a standard *Thrb* whole gene index) was incapable of revealing this striking developmental switch of TRβ2 and TRβ1 expression (see *Thrb* in the heatmap in **Figure 1B**).

We investigated these retinal datasets for other genes that serve as indicators of thyroid hormone signaling (**Figure 1D**). The *Thra* thyroid hormone receptor gene displayed modest increases in development. Previous northern blot analysis showed that the TRα1 receptor and a non-receptor splice variant α2 were both expressed with α2 in greater abundance in retina (3) as in other tissues (21, 22). Certain transporters that convey thyroid hormones across the plasma membrane, including *Slc16a2* (*Mct8*), *Slc7a5* and *Slc7a8* (43, 44) showed shifting patterns, suggesting that the control of ligand uptake or release may change during retinal maturation. Type 3 (*Dio3*) and type 2 (*Dio2*) deiodinases inactivate and activate thyroid hormone, respectively. The expression of *Dio3* and *Dio2* decreased and increased, respectively, consistent with previous findings, supporting the view that the retina progresses from a protected to a T3-sensitive state during maturation (45).

Given the sensitivity of cones to thyroid hormone, we analyzed these indicator genes in high resolution datasets for isolated cones (4), which suggested cell type selectivity compared to whole retina. For example, transcriptional coactivators (*Ncoa1*, *Ncoa2*) and corepressors (*Ncor1*, *Ncor2*) for thyroid hormone receptors were generally expressed in retina but in cones were more selective with *Ncoa1* and *Ncor1* being more prominently expressed. Expression of thyroid hormone transporters was more restricted in cones than whole retina although some were enriched such as the organic anion transporter *Slco4a1*. *Dio2* and *Dio3* expression was undetected in cones, supporting previous evidence that these genes are primarily expressed in surrounding cell types rather than the cone itself (45). In summary, the results show that TRβ1 expression rises during a phase of retinal maturation when sensitivity to thyroid hormone is acquired or refined and when systemic thyroid hormone levels rise in development (45).

### TRβ1 and TRβ2 profiles in human retinal development

To investigate similarities in mouse and human retina, we analyzed TRβ1 and TRβ2 expression using published human RNA-seq resources (**Figure 1C**, columns on *right*). Previous studies of human retina have reported general *THRB* reads without distinguishing TRβ1 and TRβ2 (9, 29, 31). We analyzed datasets representing fetal days 52 to 136 (~8 to ~20 fetal weeks) (32) and adults at 47 to 86 years of age (33). Cones are generated during the first trimester of human gestation and express S opsin (encoded by *OPN1SW*) by ~10 fetal weeks followed by L/M opsins (encoded by the *OPN1LW-OPN1MW* locus) (46). The onset of S then L/M opsin expression resembles that of S followed by M opsin in mouse development (**Figure 1D**). The maturation of opsin patterning may continue at least until birth (~40 weeks) and eye opening in humans (29, 32, 46). Morphological and functional maturation of cones continues into infancy in humans (47, 48).

Analysis of TRβ2- and TRβ1-specific exons identified a peak of TRβ2 expression at human fetal weeks ~8 to 18 (days 52 - 136) then low levels at postnatal, adult ages. In contrast, TRβ1 mRNA levels rose as TRβ2 declined during later fetal development. At adult ages, expression of TRβ1 was maintained whereas TRβ2 remained low. Although the time course is prolonged in human development, the overall trend resembled that in the mouse or chick (24) with sequential peaks of TRβ2 followed by TRβ1.

**Figure 2:**
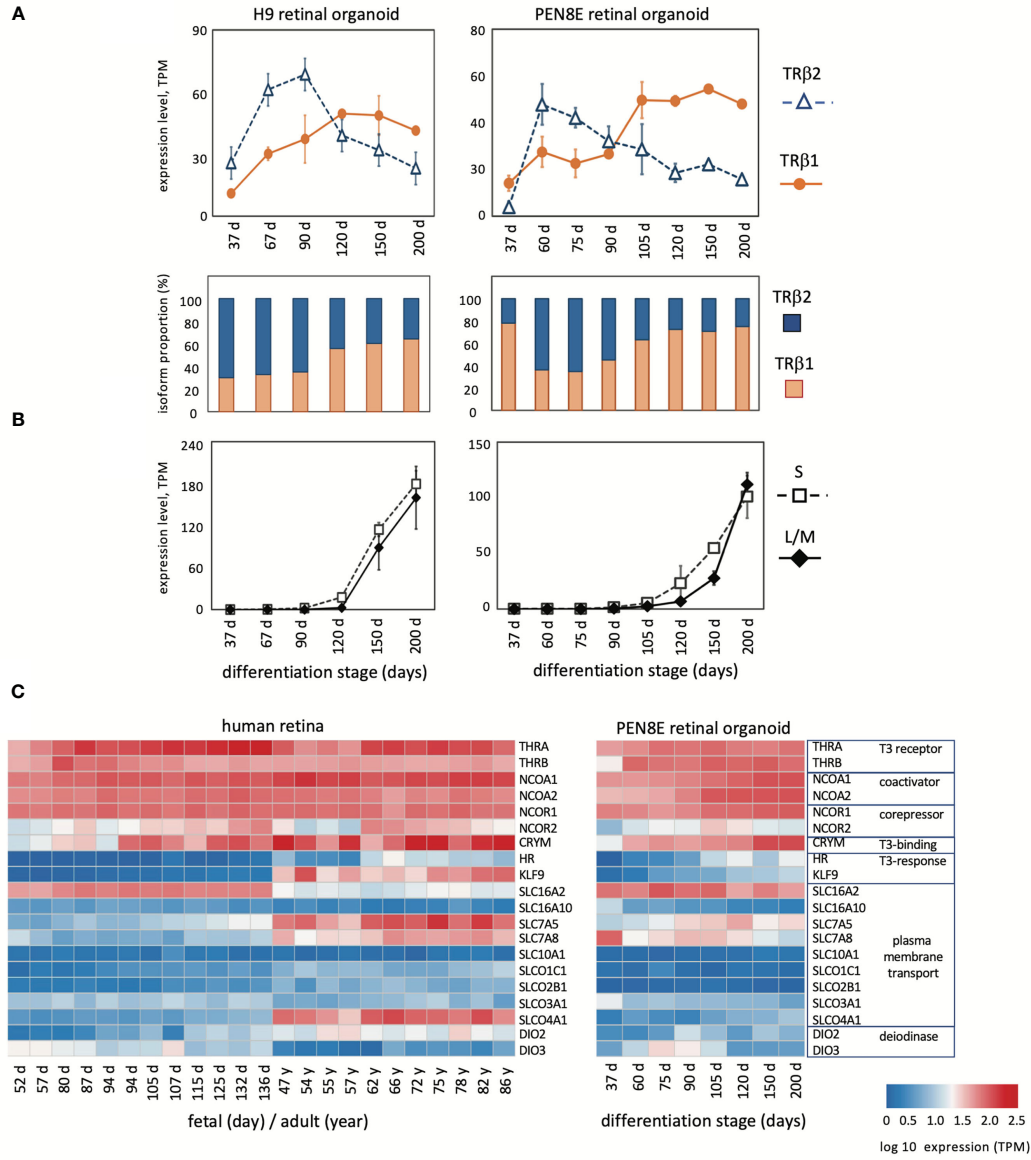
Expression of *THRB* and other genes in human retinal organoids and retina. **(A)**, RNA-seq analysis of TRβ1 and TRβ2 in H9 organoids and PEN8E organoids, derived from embryonic stem cells and iPSC lines, respectively. Mean ± S.D. The relative proportion of each isoform is indicated in the column graph, below. **(B)**, Cone opsin expression in the same RNA-seq datasets. *OPN1SW*, blue (S) opsin and *OPN1LW-OPN1MW*, red/green (L/M) opsins; the L/M curve represents reads from both genes in the *OPN1LW-OPN1MW* gene cluster. **(C)**, Heatmaps of expression of indicator genes for thyroid hormone signaling in human retina and PEN8E retinal organoids.

Given the interest in retinal organoids as a model for human retina, we investigated *THRB* isoform expression using RNA-seq datasets from human retinal organoids derived from iPSC line PEN8E (34, 35) and H9 embryonic stem cells (28, 35) over a period of ~37 to 200 days in culture (**Figure 2A**). During differentiation in culture, retinal organoids produce both rod- and cone-like cells and acquire a partly laminated retinal-like structure. These organoids also express opsins although the lag between onset of S and L/M opsins is short compared to human or mouse retinal tissue (**Figure 2B**). We found that both lines of retinal organoids displayed an early peak of TRβ2 at ~60 - 90 days and a lagging peak of TRβ1 at ~90 - 200 days in culture. This biphasic pattern of isoform expression resembled that in retinal tissue, suggesting that the organoid model recapitulates *THRB* expression patterns that occur in the retina.

Analysis of indicator genes for thyroid hormone signaling revealed a broadly similar pattern of dynamic developmental changes correlating with the switch of *THRB* isoforms in human retina as in mouse retina (**Figure 2C**). Retinal organoids also showed general similarities, although for some genes, such as transporter genes, expression was more restricted than in retinal tissue. The results suggest that TRβ1 expression rises in the retina and in organoid model systems during a period when sensitivity to thyroid hormone signaling is acquired or refined during tissue maturation.

### Isolation of coding cDNAs for human TRβ2 and TRβ1

To confirm the expression of coding mRNAs for TRβ2 and TRβ1 in human retinal-like tissue, we isolated full-length cDNA clones from PEN8E retinal organoids. The TRβ1 cDNA sequence encodes a protein of 461 amino acids and aligns with multiple human TRβ1 sequences in the GenBank database (49). TRβ2 is a rare isoform and is absent in most tissues. The human sequence is represented in GenBank by a single, partial 5’-fragment (471 base cDNA, pituitary adenoma origin, GenBank X74497) but no full-length cDNA or published reference. In rodent models, TRβ2 is detected in very few tissues (e.g. pituitary, cochlea, retina) (21, 41). The full-length human TRβ2 cDNA we isolated encodes a 476 amino acid protein as predicted from the human *THRB* gene exons and aligns with the mouse TRβ2 cDNA (30).

### Localization of TRβ1 in the neural retina

**Figure 3:**
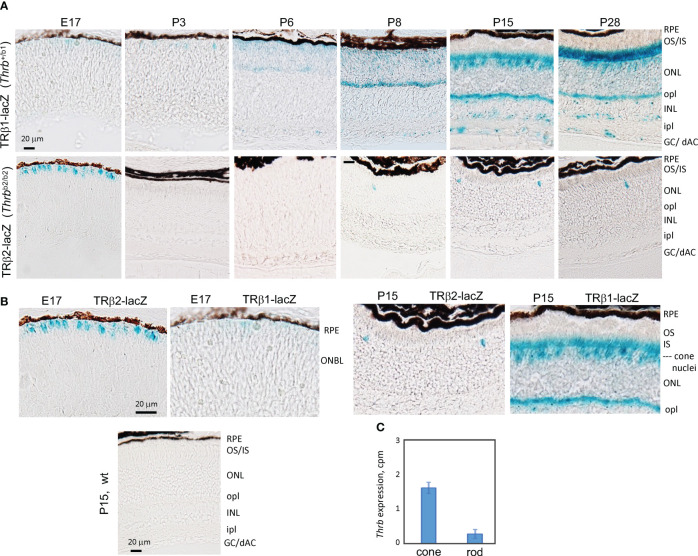
Developmental expression of TRβ1 in the retina detected in *Thrb* ^b1^ lacZ reporter mice. **(A)**, TRβ1 detected as β-galactosidase activity using x-gal substrate (blue) in cryosections from *Thrb^+/^* ^b1^ reporter mice. TRβ2 was detected using *Thrb* ^b2/b2^ lacZ mice. X-gal color reaction times were overnight (~16 hr) except at P28 for *Thrb* ^+/b1^, with a time of 6 hr to avoid saturation of signal. At least 6 retinas (n ≥ 3 mice) analyzed per stage. **(B)**, Higher magnification showing the shift from TRβ2 to TRβ1 expression in cone differentiation. Wild type (wt) sections gave little or no background. **(C)**, RNA-seq detection of *Thrb* (total gene) expression in isolated cones and rods; mean ± S.D., 34 cones, 29 rods; 2 month old mice. GC/dAC, ganglion cell/displaced amacrine cell layer; INL, inner nuclear layer; ipl, inner plexiform layer; IS, inner segments; ONBL, outer neuroblastic layer; ONL, outer nuclear layer; opl, outer plexiform layer; OS, outer segments, RPE, retinal pigmented epithelium.

To corroborate the expression profiles revealed by RNA-seq analysis and to localize TRβ1 in the retina, we analyzed *Thrb* ^b1^ reporter mice with a *lacZ* knockin at a TRβ1-specific exon in the endogenous *Thrb* gene (23). In the embryonic retina, we detected only occasional, weakly *lacZ*-positive cells in the outer neuroblastic layer where newly-generated, immature cones reside (**Figures 3A, B**). After P3, signals rose in this outer zone of the outer nuclear layer (ONL) during retinal maturation. By P15, shortly after eye-opening, signals increased, filling the cone soma, pedicles that contact inner retinal interneurons and the light-detecting segments. We also detected signals in sub-populations of neurons in the inner nuclear layer (INL) and ganglion cell/displaced amacrine cell layer at postnatal ages. In contrast, TRβ2, detected using *Thrb* ^b2^ *lacZ* reporter mice, was restricted to cones and peaked at late embryonic stages as reported (3). The biphasic peaks of TRβ2 and TRβ1 correlated closely with the RNA-seq results.

During postnatal maturation, very weak signals for TRβ1 were detected over the width of the ONL which is composed primarily of rods, the photoreceptors that mediate vision in dim light. In mice, rods outnumber cones ~30-fold and occupy most of the ONL whereas the sparser cone nuclei reside at the outer edge of the ONL (50). Further analysis of RNA-seq data from isolated rods and cones (4) detected only low *Thrb* reads in rods (**Figure 3C**), consistent with very low TRβ1 expression in rods compared to cones.

**Figure 4:**
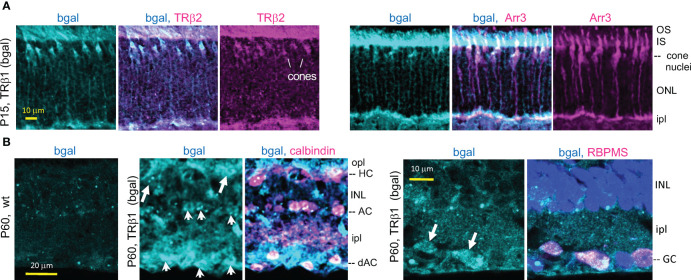
TRβ1 localization in cones and inner retinal neurons detected in *Thrb* ^b1^ lacZ reporter mice. **(A)**, TRβ1 (β-galactosidase, bgal protein immunofluorescence) co-staining with cone markers (TRβ2, Arr3) in *Thrb^+/^* ^b1^ mice. **(B)**, TRβ1 (bgal) in amacrine (arrowheads) and horizontal cells (arrows) shown by staining for calbindin (*left*). Staining was sometimes weak and appeared punctate. TRβ1 was also detected in ganglion cells (arrowheads) by staining for RBPMS (*right*). In panel **(B)**, *Thrb* ^b1/b1^ homozygotes were analyzed to enhance detection of bgal. For RBPMS, a general nuclear stain (DAPI, blue) shows tissue background. AC, amacrine cell; dAC, displaced AC; GC, ganglion cell; HC, horizontal cell; INL, inner nuclear layer; ipl, inner plexiform layer; IS/OS, inner/outer segments; ONL, outer nuclear layer; opl, outer plexiform layer.

We confirmed expression of TRβ1 (as β-galactosidase protein encoded by *lacZ*) in cones by double-staining with cone markers in *Thrb* ^+/b1^ lacZ reporter mice (**Figure 4A**). Immunofluorescence at P15 identified β-galactosidase-positive cones that co-stained for TRβ2, indicating co-expression of both TRβ isoforms during cone maturation. The cone identity was further confirmed by co-staining for cone arrestin (Arr3).

The *lacZ* staining pattern in the inner retina in *Thrb* ^+/b1^ mice suggested expression in amacrine cells which are involved in processing signals relayed from the photoreceptors to the ganglion cells. Amacrine cells exist as many sub-types based on staining with markers such as calbindin and laminar location (51). β-galactosidase-positive cells detected in both the inner zone of the INL and the displaced amacrine cell zone (in the ganglion cell layer) co-stained with calbindin (**Figure 4B**) indicating TRβ1 expression in amacrine cell populations. Calbindin also stains horizontal cells. β-galactosidase-positive cells in the horizontal cell layer of the INL stained with calbindin, indicating TRβ1 expression in horizontal cells. We investigated if β-galactosidase signals in the ganglion/displaced amacrine cell layer also localized in ganglion cells by staining with RBPMS, a ganglion cell marker (52). β-galactosidase-positive cells co-stained with RBPMS, indicating expression of TRβ1 in ganglion cells.

### TRβ1 in the ciliary margin zone, ciliary body, iris and retinal pigmented epithelium

**Figure 5:**
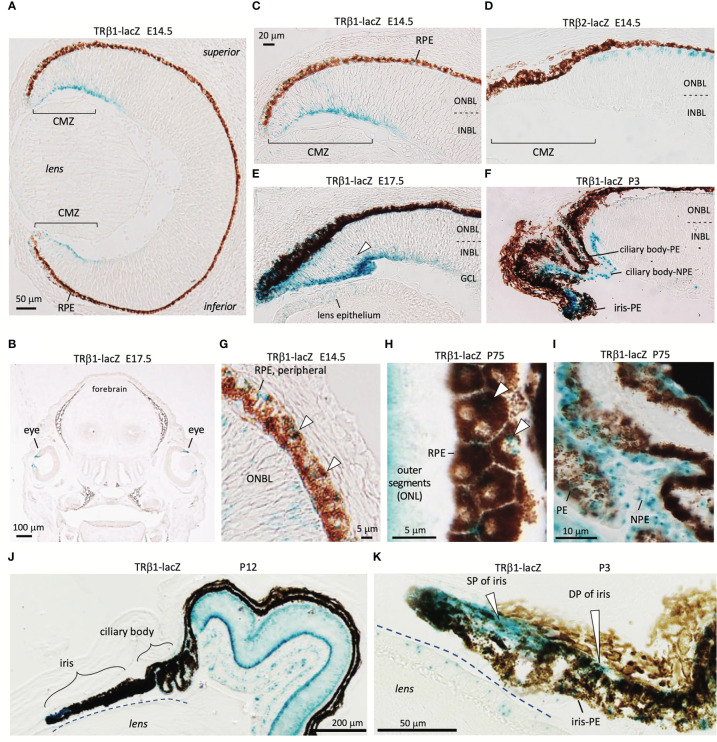
TRβ1 in the ciliary margin zone, iris and associated tissues detected in *Thrb* ^b1^ lacZ reporter mice. **(A, B)**, TRβ1 in the ciliary margin zone (CMZ) (x-gal staining, blue) in *Thrb* ^+/b1^ embryos. Otherwise, expression is rare in embryonic tissues (coronal head section in *B*). **(C, D)**, Expression of TRβ1 in the CMZ contrasts with TRβ2 in cone precursors shown in *Thrb* ^b2/b2^ lacZ reporter mice. **(E)**, TRβ1 in the late embryonic CMZ. Arrowhead, positive progenitor cells in the neuroblastic layers of the CMZ. Weak signals are detected in the lens epithelium. **(F)**, TRβ1 in the non-pigmented (NPE) and pigmented epithelia (PE) of the ciliary body at P3. **(G, H)**, TRβ1 in the retinal pigmented epithelium (RPE) (arrowheads) at embryonic **(G)** and mature **(H)** stages. Oblique section in *H* shows the planar polygonal shape of RPE cells. **(I)**, TRβ1 expression in NPE and PE of the ciliary body in the adult. **(J, K)**, Iris and ciliary body at P12 in overview **(J)** and iris at P3 at higher magnification **(K)**. TRβ1 is detected in the sphincter pupillae (SP) and dilator pupillae (DP) and pigmented epithelium of the iris (iris-PE). CMZ, ciliary margin zone; GCL, ganglion cell layer; INBL, inner neuroblastic layer; NPE, non-pigmented epithelium of ciliary body; ONBL, outer neuroblastic layer; ONL, outer nuclear layer; PE, pigmented epithelium of ciliary body; RPE, retinal pigmented epithelium.

TRβ1 is undetectable in most embryonic tissues but was identified in the early ciliary margin zone (CMZ) and associated anterior structures of the eye in *Thrb* ^+/b1^ reporter mice at E14.5 (**Figures 5A–I**). The CMZ gives rise to non-neural epithelia of the ciliary body (CB) and iris (53) and has been reported to have a potential to generate some neurons that contribute to the neural retina (54). The expression of TRβ1 in the early CMZ was in striking contrast to TRβ2 in newly-generated cone precursors (**Figures 5C, D**), indicating radically different control of the TRβ1 and TRβ2-specific promoters of the *Thrb* gene by different cell types in retinal development.

The ciliary body, which supports lens focusing, acquires a folded morphology in mouse postnatal development (53)(**Figures 5F, I**). TRβ1 was detected in the pigmented epithelia of the ciliary body as well as the non-pigmented cells that produce the aqueous humor of the eye. The iris expressed TRβ1 in both the sphincter pupillae and dilator pupillae, muscles that control the aperture opening of the pupil (**Figures 5J, K**). TRβ1 was also detected in the retinal pigmented epithelium (RPE), which provides support for photoreceptors (**Figures 5G, H**). These results suggest wider roles for TRβ1 in non-neural ocular tissues as well as in photoreceptors and neurons of the inner retinal layers.

### Cone gene expression in TRβ1-deficient mice

**Figure 6:**
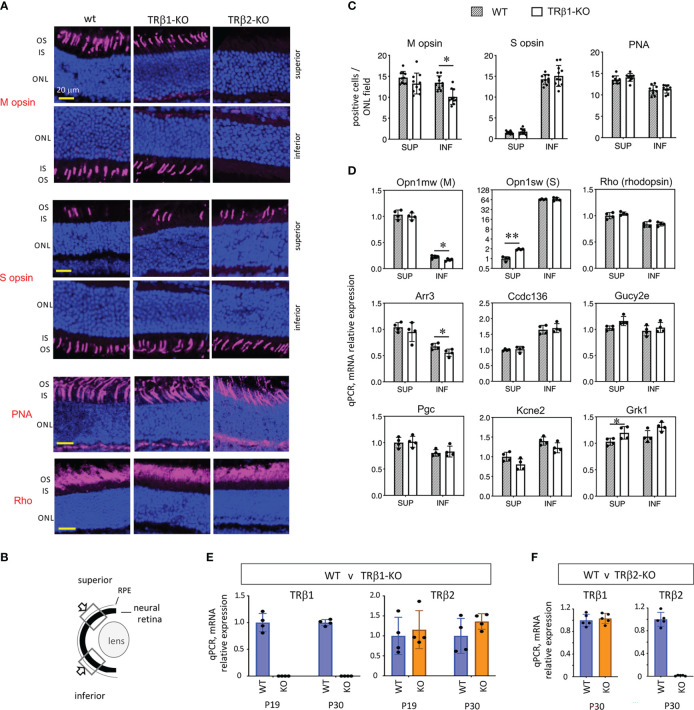
Cone gene expression in TRβ1-KO mice. **(A, B)**, Opsin immunostaining (magenta, in outer segments) in retinal cryosections showing superior and inferior views in control (wt) and TRβ1-KO (*Thrb* ^b1/b1^) adult mice (~2 months old). Fields of view represent 100 μm lengths of ONL (for locations see *B)*. For comparison, a representative TRβ2-KO displays severe loss of M opsin and extended expression of S opsin in all areas. PNA, peanut agglutinin, pan-cone marker. Rho, rod photopigment, rhodopsin. Dark blue, nuclear (DAPI) stain, shows tissue background. **(C)**, Counts of opsin- and PNA-positive cones. Mean ± S.D., adult mice; 10 views from the superior and 10 from the inferior retina in the vertical plane around the mid-retinal region obtained from 6 retinas from 3 mice; *p < 0.05, unpaired t-test. **(D)**, Cone and rod gene mRNA expression analyzed by qPCR. Control mice display counter-gradients of M (*Opn1mw*) and S (*Opn1sw*) opsin mRNA over the superior - inferior plane. In TRβ1-KO mice, *Opn1mw* and *Arr3* are marginally decreased in the inferior retina and *Opn1sw* modestly elevated in the superior. Most genes show little or no change. Groups, n = 4 mice, with 2 retinas pooled per mouse per region; mean ± S.D., *p < 0.05; **p < 0.001, unpaired t-test on log transformed expression data. **(E)**, TRβ2 mRNA expression is unchanged in TRβ1-KO mice shown at 2 ages (mean ± S.D., p = 0.58 (P19) and p = 0.16 (P30), unpaired t-test, log transformed expression data); n = 4 retinas representing at least one retina from each of 3 mice. TRβ1-KO lacks TRβ1 mRNA as expected. **(F)**, TRβ1 mRNA expression is unchanged in TRβ2-KO mice, shown at P30 (mean ± S.D., p = 0.61, unpaired t-test on log transformed expression data); n = 5 retinas representing at least one retina from each of 3 mice. ONL, outer nuclear layer; OS/IS, outer/inner segments.

The expression of TRβ1 in cones led us to test a role for TRβ1 in opsin regulation by investigation of TRβ1-KO mice. In mice at mature ages, M and S opsins are expressed in counter-gradients over the superior-inferior plane of the retina. M opsin has a modest gradient biased to the superior and S opsin has a more marked gradient biased to the inferior (**Figures 6A, B**) (1). Opsin expression changes, if pronounced, may be detected by immunostaining on retinal sections. TRβ1-KO adult mice (~2 months old) displayed only marginal decreases of M opsin-positive cones in inferior regions and no obvious change of S opsin-positive cones (**Figure 6C**). In comparison, TRβ2-KO mice have severe loss of M opsin in all regions and S opsin expression extends to all cones (example in **Figure 6A**) (3). TRβ1-KO mice retained normal total cone numbers, indicated by staining with peanut agglutinin (PNA), a pan-cone marker (**Figure 6C**). These results were corroborated by qPCR analysis of *Opn1mw* (M) and *Opn1sw* (S) mRNA in isolated pieces of superior and inferior retina (**Figure 6D**). Expression of *Opn1mw* was slightly decreased in inferior regions whereas *Opn1sw* was moderately increased in superior regions. These minor changes in TRβ1-KO mice resemble in a minimal way the extreme phenotypes in TRβ2-KO mice, suggesting that TRβ1 may influence some similar transcriptional pathways as TRβ2 in cones.

Other TRβ2-regulated cone genes (4) displayed only limited changes in TRβ1-KO mice. Expression of cone arrestin (*Arr3*), which has an M opsin-like superior bias, was slightly decreased in the inferior retina, partly resembling the defect in TRβ2-KO mice (4). Other TRβ2-dependent genes including *Pgc*, *Ccdc136* and *Kcne2* showed no obvious change in TRβ1-KO mice. These results suggest that TRβ1 has a limited contribution to the control of TRβ2-dependent genes. No obvious changes were detected in expression of rhodopsin, the rod photopigment, by immunostaining or by qPCR analysis of *Rho* mRNA in TRβ1-KO mice. Selected phototransduction genes common to cones and rods showed little (*Grk1*) or no (*Gucy2e*) change in expression.

We investigated TRβ2 mRNA in retina by qPCR to assess if elevation of TRβ2 levels might compensate for loss of TRβ1 in TRβ1-KO mice (**Figure 6E**). As expected TRβ1 levels were severely depleted in TRβ1-KO mice at P19 or P30. However, TRβ2 mRNA levels were similar in wild type and TRβ1-KO mice. If there is compensation by TRβ2, this would presumably be accomplished by TRβ2 levels in the normal range. In a similar analysis, we found no obvious distortion of TRβ1 expression in TRβ2-KO mice (**Figure 6F**).

### Electroretinogram analysis

**Figure 7:**
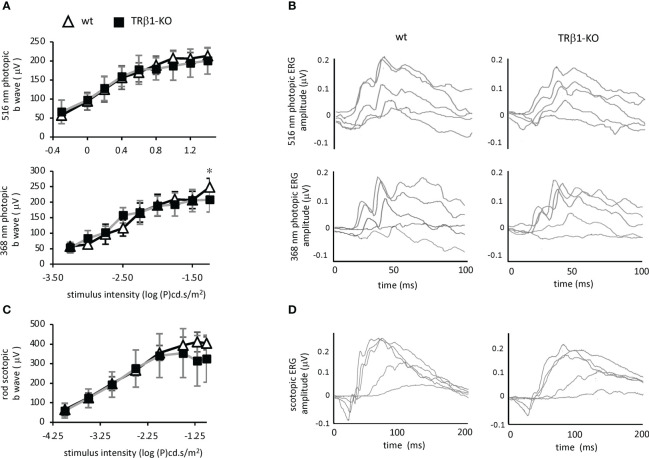
Electroretinogram analysis of TRβ1-KO mice. **(A)**, Photopic, light-adapted responses to optimal wavelengths for stimulation of cone M opsin (516 nm) and S opsin (368 nm) in mice. Curve plots show b-wave magnitudes, mean ± S.D. Groups, 6 wild type and 8 TRβ1 KO mice, ~2 months of age. * p < 0.05, unpaired t-test for each stimulus intensity. **(B)**, Example waveforms in response to varying stimulus intensities in single representative mice; intensities of 1.26, 3.16, 10, 19.95 and 32 cd.s/m^2^ for 516 nm; 0.0001, 0.001, 0.0031, 0.01 and 0.0316 cd.s/m^2^ for 368 nm. **(C)**, Scotopic, dark-adapted responses indicating rod function, showing b wave magnitudes, mean ± S.D. Groups, 8 wt and 7 TRβ1 KO mice, 6 - 8 weeks old. **(D)**, Example waveforms for varying stimulus intensities of 0.0001, 0.001, 0.01, 0.0316 and 0.1 cd.s/m^2^ for scotopic responses.

To investigate cone function, we analyzed the photopic electroretinogram in TRβ1-KO adult mice. Responses to light stimuli at 516 nm and 368 nm, optimal wavelengths for stimulation of M and S opsins, respectively in mice (55), were in the normal ranges in TRβ1-KO mice (**Figure 7**). The b-wave magnitudes were in the normal range in TRβ1-KO mice. Rod responses in scotopic, dark-adapted electroretinogram analyses, lacked obvious defects in TRβ1-KO mice. These results indicate that unlike deletion of TRβ2 (3), deletion of TRβ1 in mice does not result in obvious defects in cone function.

## Discussion

We report a novel pattern of TRβ1 expression in ocular tissues, suggesting remarkably diverse roles for thyroid hormone in cones, other retinal neurons and non-neural cell types. The findings indicate the importance of specific analyses of individual receptor isoforms encoded by *Thrb* rather than general analyses of *Thrb* expression in total. Our results suggest that the *Thrb* gene accomplishes diverse functions in the eye by independent regulation of two promoters that drive differential expression of the TRβ1 and TRβ2 receptor isoforms. The similar temporal switches of TRβ1 and TRβ2 in mouse and human retina suggest conserved functions during maturational periods when sensitivity to thyroid hormone is determined.

### Biphasic expression of TRβ isoforms in the neural retina

In mice, cones mature into M and S opsin-expressing populations during postnatal development (42). Surprisingly, although TRβ1 is expressed in cones, only subtle opsin changes were detected in TRβ1-KO mice unlike the extreme loss of M opsin and gain of S opsin in TRβ2-KO mice (3). It is possible that the persistent low levels of TRβ2 at mature ages suffice to minimize cone phenotypes in TRβ1-KO mice. However, the converse is not true; i.e., TRβ1 cannot compensate for deletion of TRβ2. This might be explained if TRβ2 primes gene expression in immature cones in a way that cannot be achieved by the later expression of TRβ1 when the epigenetic status of the cone lineage may be less pliable (4). In support of this proposal, we recently demonstrated that an intronic enhancer in the *Thrb* gene determines the appropriate timing and level of expression of endogenous TRβ2 protein and consequently, levels of M opsin and the spectral sensitivity of cones (38). The timing and cell-specificity of TRβ2 expression suggest that a threshold level of receptor at a sensitive developmental time is required for normal maturation and function of cone photoreceptors.

Mutations of the human *THRB* gene have been associated with monochromacy and impaired spectral sensitivity in the syndrome of resistance to thyroid hormone (6–8). All known *THRB* mutations in this syndrome occur in common regions of the gene and interfere with both TRβ1 and TRβ2 (56). Mutation of the *THRB* common region in human retinal organoids impairs expression of the *OPN1LW-OPN1MW* locus (9). In the organoid model study, the possibility was raised that TRβ1 activates the *OPN1LW-OPN1MW* locus although a specific deletion of TRβ1 has not been reported. The sequential peaks of TRβ2 then TRβ1, first observed in the chick, are broadly similar in human and mouse development and in human retinal organoids (**Figure 1**). However, the full course of cone differentiation in the context of the human lifespan is prolonged over many months and extends into infancy (46, 47). It is unclear if the TRβ2 and TRβ1 peaks might overlap during this period to a greater extent in human than mouse retinal development. Further insights may be gained by localization of TRβ1 in human retinal cell types. Immunostaining previously detected TRβ2 in human fetal cones and in human retinoblastomas, which arise from cone-like L/M opsin-positive cells (57).

It is not excluded that TRβ1 might modify responses to challenge or stress at adult or aging stages. Prolonged hypothyroidism when induced in adult rodents over months can reversibly alter M and S opsin patterning (58). This residual plasticity of patterning might reflect latent function of the normal developmental program and could involve TRβ1, the predominant TRβ isoform at mature ages. TRβ1 might also contribute to cone loss caused by thyroid hormone excesses (15, 16). Deletion of the *Thrb* gene diminishes cone loss in models of retinal degeneration (19) and reduces loss of RPE and photoreceptors in a chemically-induced model of macular degeneration (20), which might involve TRβ1 at mature ages.

The detection of TRβ1 in the inner retina suggests possible roles for thyroid hormone in amacrine cells, which process visual information transmitted from the photoreceptors, and in the ganglion cells that relay these signals through the optic nerve (51, 59). Consistent with our results, studies of a mouse reporter model (FIND-T3) for thyroid hormone activity suggested amacrine and ganglion cells as targets in the postnatal retina (60). This may suggest a role for thyroid hormone in visual processing in the inner retina but this remains to be explored. It has been reported that hypothyroidism impairs visual-evoked potentials in central pathways in rats (13, 61).

### TRβ1 in the ciliary body, iris and retinal pigmented epithelium

TRβ1 is undetected in most embryonic tissues but is detected in the ciliary margin zone and anterior tissues that give rise to the ciliary body and iris of the eye (53). The iris controls the aperture of the pupil and entry of light whereas the ciliary body focuses the lens and also produces aqueous humor. The ciliary margin zone can also generate some neurons that contribute to the neural retina (54, 62). There has been little study of thyroid hormone action in these tissues but the TRβ1 expression pattern suggests possible functions in anterior structures of the eye.

We also detected TRβ1 in the RPE, which provides crucial support for the photoreceptors. Other studies have reported thyroid hormone receptor gene expression in human RPE cells in culture (63) and in the RPE of zebrafish (64). The RPE mediates transepithelial transport and is involved in the renewal of outer segment discs, the opsin-packed structures of photoreceptors. Material from disc shedding is phagocytosed by the RPE. Hypothyroidism in adult rats has been reported to reduce the rate of disc renewal (65). It is noteworthy that deletion of *Thrb* can protect RPE cells and photoreceptors from damage in a chemically-induced model of macular degeneration (20). Our findings suggest that this susceptibility to damage might reflect TRβ1 functions in both RPE and cone cells. In amphibian and fish species, a function of thyroid hormone is to induce cyp27c1 in the RPE, which produces vitamin A2, a specialized chromophore for enhanced sensitivity to red light (66).

Severe developmental hypothyroidism in rats retards craniofacial features such as width of the eye-nose axis, eyeball mass, opening of the eyelids and thickening of retinal layers (67–69). Although this retardation has undefined cellular etiology, it is possible that TRβ1 underlies some of these functions in the eye. The *Thra* gene is also expressed in the retina (24) and RPE (64) suggesting that the TRα1 receptor mediates functions in the eye that remain to be discovered. In summary, our finding of differential expression of TRβ1 and TRβ2 reinforces the view that ocular development is coordinated in part by thyroid hormone acting on specific retinal and non-neural cell types in the eye.

## Data availability statement

The original contributions presented in the study are included in the article/supplementary material. Further inquiries can be directed to the corresponding author.

## Ethics statement

The animal study was reviewed and approved by NIH/NIDDK Animal Care and Use Committee.

## Author contributions

LN, HL, YL and DF designed research. LN, HL and YL performed research. LN, HL, YL and DF analyzed data. DF, LN wrote the paper with input from all authors. All authors contributed to the article and approved the submitted version.
